# Identification of a Critical and Conformational Neutralizing Epitope in Human Adenovirus Type 4 Hexon

**DOI:** 10.1128/JVI.01643-17

**Published:** 2018-01-02

**Authors:** Xingui Tian, Hongling Qiu, Zhichao Zhou, Shouli Wang, Ye Fan, Xiao Li, Ruiai Chu, Haitao Li, Rong Zhou, Hui Wang

**Affiliations:** a State Key Laboratory of Respiratory Disease, National Clinical Research Center for Respiratory Disease, Guangzhou Institute of Respiratory Disease, the First Affiliated Hospital of Guangzhou Medical University, Guangzhou Medical University, Guangzhou, China; b Key Laboratory of Food Safety, Shanghai Institutes for Biological Sciences, Chinese Academy of Sciences, Shanghai, China; c State Key Laboratory of Quality Research in Chinese Medicine, University of Macau, Taipa, Macao, China; d Institute of Chinese Medical Sciences, University of Macau, Taipa, Macao, China; University of California, Irvine

**Keywords:** adenovirus type 4, conformational epitope, neutralizing monoclonal antibody, acute respiratory disease, adenoviral vector

## Abstract

Human adenovirus type 4 (HAdV-4) is an epidemic virus that contributes to serious acute respiratory disease (ARD) in both pediatric and adult patients. However, no licensed drug or vaccine is currently available to the civilian population. The identification of neutralizing epitopes of HAdV-4 should allow the development of a novel antiviral vaccine and a novel gene transfer vector, and an effective neutralizing monoclonal antibody (MAb) will be useful in developing appropriate antiviral drugs. In this study, we report that MAb MN4b shows strong neutralizing activity against HAdV-4. MN4b recognizes a conformational epitope (418AGSEK422) within hypervariable region 7 (HVR7). Mutations within this site permitted HAdV-4 mutants to escape neutralization by MN4b and to resist neutralization by animal and human anti-HAdV-4 sera. A recombinant virus, rAd3-A4R7-1, containing the identified neutralizing epitope in the HVR7 region of HAdV-3 hexon, successfully induced antiserum that inhibited HAdV-4 infection. These results indicate that a small surface loop of HAdV-4 hexon is a critical neutralization epitope for this virus. The generation of MN4b and the identification of this neutralizing epitope may be useful in developing therapeutic treatment, a subunit vaccine, and a novel vector that can escape preexisting neutralization for HAdV-4.

**IMPORTANCE** Neutralizing antibodies are considered good tools for the prevention of human adenovirus type 4 (HAdV-4) infections. The identification of the epitopes recognized by such neutralizing antibodies is important for the generation of recombinant antiviral vaccines. However, until now, no neutralizing epitope has been reported for HAdV-4. Here, we developed a serotype-specific neutralizing MAb directed against HAdV-4, MN4b. We provide evidence that MN4b recognizes a conformational epitope within HVR7 of HAdV-4 hexon. Antisera generated to this conformational epitope displayed on HAdV-3 hexon inhibited infection of AD293 cells by HAdV-4. Our findings are very important for the development of therapeutic treatment, a subunit vaccine, and a novel vector for HAdV-4.

## INTRODUCTION

Human mastadenoviruses (HAdVs) are highly contagious pathogens that cause a broad spectrum of diseases in children and adults, including acute respiratory infections, acute gastroenteritis, epidemic keratoconjunctivitis, and genitourinary illnesses (1–3). To date, more than 81 genotypes are recognized, which are divided into seven species (species A to G) (4–6). The genotypes of specific species are often associated with particular clinical manifestations. HAdV species C (HAdV type 1 [HAdV-1], HAdV-2, HAdV-5, and HAdV-6), species B (HAdV-3, -7, -14, -21, and -55), and species E (HAdV-4) are most commonly found in patients with acute respiratory diseases (ARDs). HAdV-4 is among the most commonly reported species and is associated with severe ARDs in both pediatric and adult patients (7–12). HAdV-4 is the principal etiological agent of ARDs in unvaccinated U.S. military trainees (13–15).

Approved live oral vaccines based on HAdV-4 and HAdV-7 derived from human diploid cells have been used in U.S. military recruits to reduce febrile respiratory illness (FRI) and have significantly reduced the risk of FRI in the U.S. military (14, 16). However, no vaccine is currently approved for general use in children and adults, and no efficient antiviral therapy for adenoviruses is available.

Neutralizing monoclonal antibodies (MAbs) are promising prophylactic or therapeutic drugs against viral disease. The generation of neutralizing MAbs is also useful in identifying neutralizing epitopes, a highly important step in the molecular design of vaccines. HAdV-4 has also been proposed as an alternative vector for human immunodeficiency virus (HIV)/simian immunodeficiency virus (SIV) and candidate influenza vaccines (17). To escape the neutralization reaction *in vivo*, the neutralizing epitope is modified in a viral gene delivery vector. Well-characterized neutralization epitopes are useful for the construction of such vectors (18–20). The adenoviral capsid is composed of three major structural proteins: hexon, penton base, and fiber. Adenovirus-neutralizing antibodies can be raised against any of these major capsid proteins (21, 22), although the hexon protein is the predominant target of serotype-specific neutralizing antibodies (NAbs) (22–27). The serotype-specific neutralization epitopes on hexon are located mainly on the tower region, which consists of seven hypervariable regions (HVRs), among which HVR7 can be further subdivided into three additional highly variable regions (28–32). The specific locations of the neutralizing epitopes of HAdV-4 have not been identified.

In this study, we report a neutralizing MAb with strong neutralizing activity against HAdV-4, MN4b, which recognizes a conformational epitope within HVR7. Neutralization assays confirmed that mutants within this site escaped neutralization by MN4b, by antisera from animals immunized with HAdV-4, and by sera from humans infected with HAdV-4. These results indicate that this epitope is a critical neutralization site in HAdV-4 hexon.

## RESULTS

### Identification of neutralizing MAb MN4b directed against HAdV-4 hexon.

**FIG 1:**
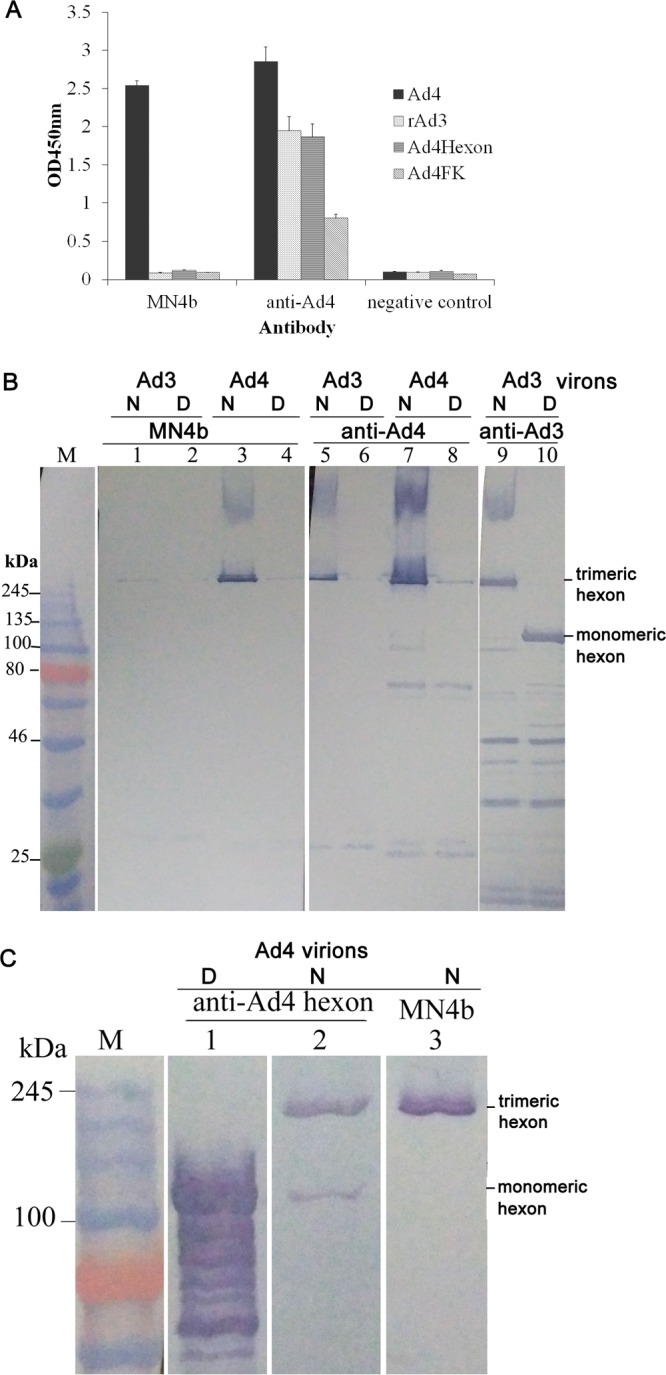
MAb MN4b recognizes HAdV-4 hexon in its trimeric form. (A) The reaction of MN4b with wild-type HAdV-4 (wAd4), rAd3, a recombinant hexon fragment (Ad4hexon), or fiber knob (Ad4FK) of HAdV-4 was detected by ELISAs. Antiserum from mice immunized with HAdV-4 (anti-Ad4) was used as the positive control, and antiserum from mice immunized with PBS was used as the negative control. Each experiment was repeated independently at least three times, and the means ± standard deviations are shown. OD450nm, optical density at 450 nm. (B) Immunoblot analysis indicates that MN4b recognizes HAdV-4 hexon in its trimeric form but not the hexon monomer. Purified HAdV-4 virions were stored at room temperature (native [N]) (lanes 3 and 7) or heated at 98°C (denatured [D]) (lanes 4 and 8) for 5 min in the presence of loading buffer. Purified HAdV-3 virions at room temperature (native) (lanes 1, 5, and 9) or boiled at 98°C (denatured) (lanes 2, 6, and 10) were used as the controls. The samples were then separated by SDS-PAGE and transferred onto polyvinylidene difluoride membranes. The membranes were incubated with MN4b (lanes 1 to 4) or anti-Ad4 antiserum (lanes 5 to 8). Purified HAdV-3 virions that disintegrated at room temperature (lane 9) or at 98°C (lane 10) were incubated with mouse anti-HAdV-3 serum as controls. (C) Immunoblot analysis confirmed that MN4b recognizes HAdV-4 hexon. Purified HAdV-4 virions were stored at room temperature (native) (lanes 2 and 3) or heated at 98°C (denatured) (lane 1) for 5 min in the presence of loading buffer. The membranes were then incubated with MN4b (lane 3) or antiserum from a mouse immunized with recombinant HAdV-4 hexon expressed in E. coli (anti-Ad4hexon) (lanes 1 and 2). M, standard prestained protein marker (NEB, UK).

Of the six anti-HAdV-4 MAbs generated, only the monoclonal IgG1 isotype antibody MN4b had high neutralizing activity against HAdV-4 in AD293 cells. The ascites titer of MN4b was determined by an enzyme-linked immunosorbent assay (ELISA) against HAdV-4 virions and was about 800,000. MN4b had a high neutralization titer of up to 3,200 (about 0.6 μg/ml) against HAdV-4. MN4b did not neutralize HAdV-3, HAdV-7, or HAdV-5, indicating that it is a serotype-specific NAb against HAdV-4 (data not shown). An indirect ELISA indicated that MN4b reacted with its parental antigen, whole HAdV-4 GZ01 virus particles, suggesting that the epitope recognized by MN4b is presented on the surface of the virions (Fig. 1A). MN4b did not react with purified HAdV-3 virions. MN4b also did not react with the recombinant HAdV-4 hexon (Ad4hexon) peptide (amino acids 112 to 491 of hexon) or the fiber knob peptide (Ad4FK) expressed in Escherichia coli (Fig. 1A).

Native and denatured Western blot analyses were used to determine whether MN4b recognizes a conformation-dependent antigen. Western blotting was performed with purified HAdV-4 particles that had been separated electrophoretically after exposure to 1% SDS at room temperature (native) (Fig. 1B, lanes 3 and 7) or at 98°C (denatured) (lanes 4 and 8). At room temperature, the hexon protein maintains its trimeric form in SDS. MN4b recognized only the native trimeric HAdV-4 antigen (Fig. 1B, lane 3) and not the denatured monomeric HAdV-4 antigen (lane 4) that had been heated to 98°C in the presence of SDS. Anti-HAdV-4 serum reacted strongly with the native HAdV-4 antigen (Fig. 1B, lane 7) but weakly with the denatured HAdV-4 antigen (lane 8). Purified native (Fig. 1B, lanes 1, 5, and 9) or denatured (lanes 2, 6, and 10) HAdV-3 virions were used as the controls. Interestingly, anti-HAdV-3 serum reacted with the native HAdV-3 antigen (Fig. 1B, lane 9) and also with the denatured HAdV-4 antigen (lane 10). The molecular weight of the antigen recognized by MN4b (Fig. 1B, lane 3) was similar to that of the major capsid protein, the hexon homotrimer, which was recognized by anti-HAdV-4 serum and anti-HAdV-3 serum (Fig. 1B, lanes 5 and 9, respectively). Further Western blot analyses with antiserum from a mouse immunized with the recombinant HAdV-4 hexon expressed in E. coli confirmed that MN4b detected the hexon homotrimer (Fig. 1C). These results suggest that MN4b recognizes a conformation-dependent epitope on the hexon homotrimer.

### Mapping the conformational epitope recognized by MN4b.

**FIG 2:**
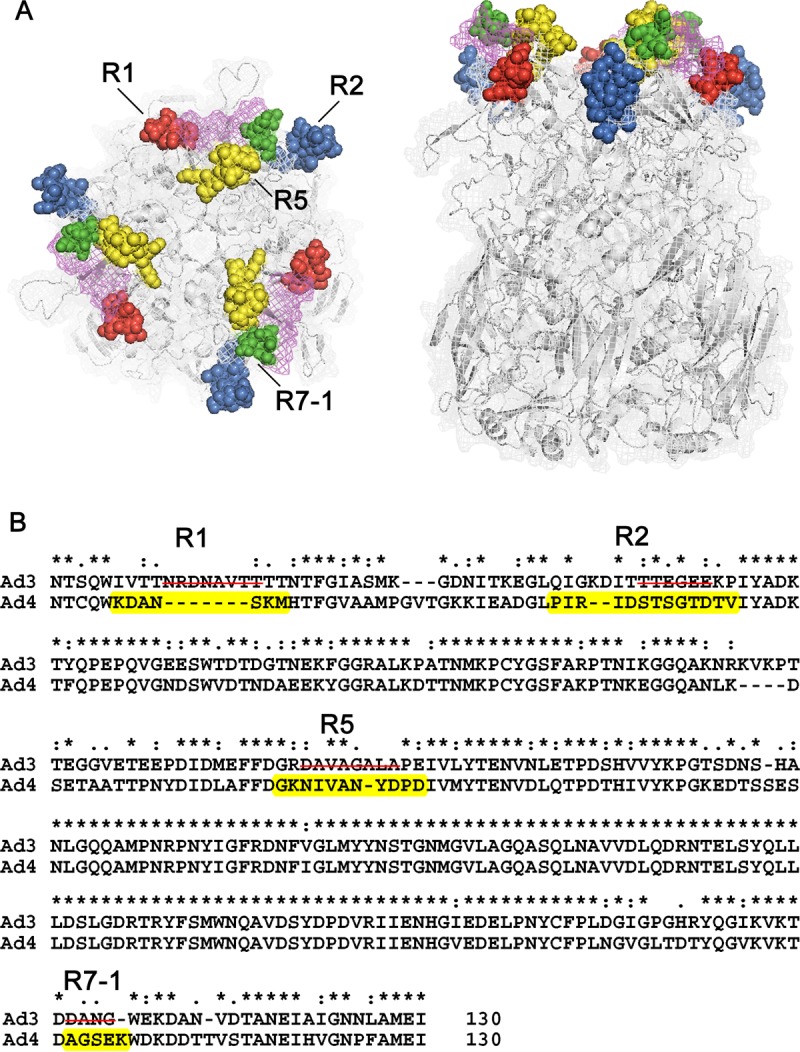
Molecular modeling and neutralizing epitope prediction for the HAdV-4 hexon. (A) Structural model of homotrimeric HAdV-4 hexon showing the potential neutralizing epitope regions. Potential epitopes are located in the four tower regions, which are shown in red (R1), blue (R2), yellow (R5), and green (R7-1) (left, top view; right, side view). (B) Partial sequence alignment of HAdV-4 hexon and HAdV-3 hexon showing residues 131 to 446 of HAdV-4 and residues 131 to 454 of HAdV-3. Amino acid residues of potential epitope regions (R1, R2, R5, and R7) are highlighted in yellow. Four rAd3EGFP-based chimeric-hexon adenoviruses were prepared by incorporating the predicted HAdV-4 epitope peptides into the corresponding surface loops of adenovirus type 3 hexon. Deleted amino acids are indicated by lines through the sequence.

Previous studies suggested that epitopes recognized by adenoviral serotype-specific NAbs are exposed on the virion surface and reside within the seven HVRs of the hexon proteins (1, 17). An HAdV-4 hexon homotrimer model was built based on template 2obe.1.A of the AdC68 hexon crystal structure (Fig. 2A). Using sequence alignment data and the constructed model of HAdV-4 hexon, four small surface loops were predicted as potential neutralizing epitopes (R1 [residues 136 to 142], R2 [residues 164 to 176], R5 [residues 256 to 267], and R7-1 [residues 418 to 422]). These potential neutralization sites were located within HVR1, HVR2, HVR5, and HVR7 of hexon, respectively (Fig. 2B).

**FIG 3:**
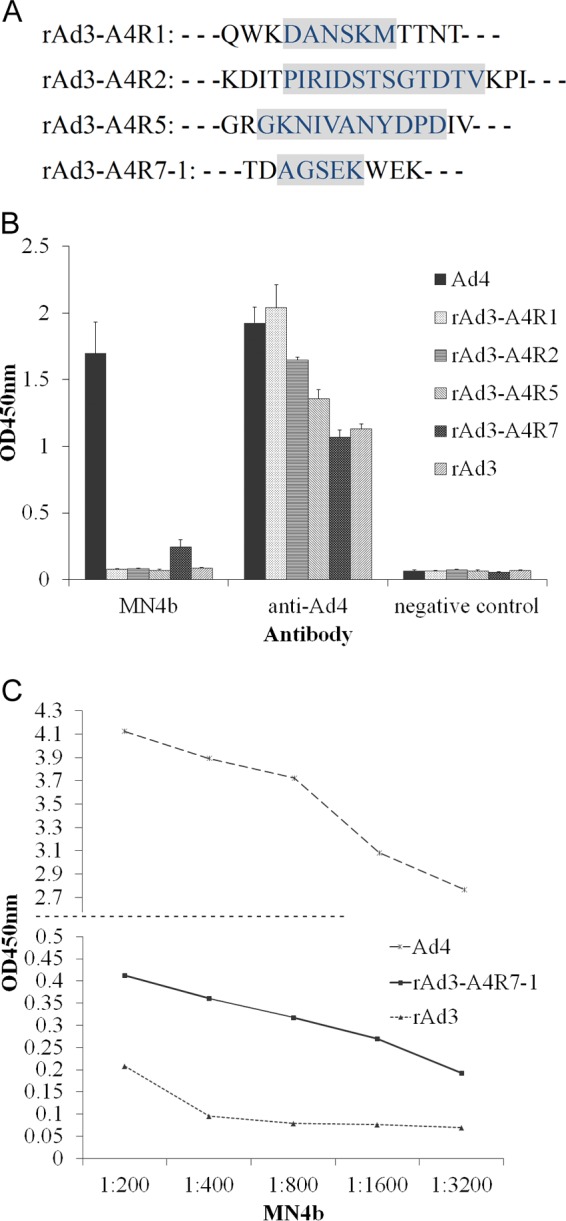
Identification of the epitope recognized by MN4b with chimeric adenoviruses displaying the potential epitopes of HAdV-4 on HAdV-3 hexon. (A) Schematic depiction of the chimeric hexon proteins in the four chimeric adenoviruses. The incorporated amino acids of the HAdV-4 epitopes are indicated in gray. (B) Indirect ELISAs were performed to assess the reactions of MN4b with a series of recombinant adenoviral particles containing chimeric hexons. Antiserum from mice immunized with HAdV-4 was used as the positive control, and antiserum from mice immunized with PBS was used as the negative control. Whole viral particles of wild-type HAdV-4 (wAd4) and rAd3 were used as the controls. Each experiment was repeated independently at least three times, and the means ± standard deviations are shown. (C) Further indirect ELISAs with serial dilutions of MN4b confirmed the reaction between MN4b and rAd3-A4R7.

Four epitope-incorporated recombinant adenoviruses were constructed by incorporating the predicted HAdV-4 epitope peptides into the corresponding surface loops of HAdV-3 hexon (Fig. 3A). These epitopes were displayed on hexon trimers assembled in virus particles. An ELISA showed that MN4b recognized rAd3-A4R7-1 but did not react with the other recombinant viruses (Fig. 3B). Further tests with serially diluted MN4b confirmed these results (Fig. 3C), which suggest that MN4b detects a conformation-dependent epitope in HVR7.

**FIG 4:**
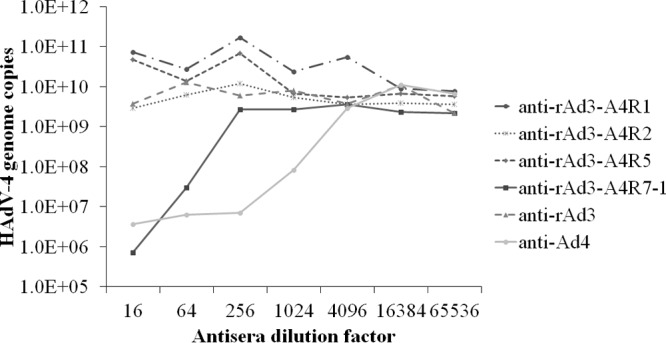
Neutralization assay of HAdV-4 with polyclonal antisera from mice immunized with epitope-chimeric adenoviruses. The antibody-virus mixtures were transferred to 96-well plates containing 85% to 95% confluent monolayers of AD293 cells. After culture for 96 h, the HAdV-4 genome copy numbers were determined by Q-PCR. Mouse anti-rAd3 and anti-Ad4 sera were used as the controls.

To determine whether these four sites contain neutralizing epitopes, antisera were generated by immunizing mice with the epitope-incorporated recombinant adenovirus rAd3-A4R1, rAd3-A4R2, rAd3-A4R5, or rAd3-A4R7-1. A neutralization test showed that only anti-rAd3-A4R7-1 serum and the positive control, anti-HAdV-4, inhibited infection of AD293 cells by HAdV-4 (Fig. 4). Therefore, only the A4R7-1 epitope induced NAbs against HAdV-4 and is an important neutralizing epitope of HAdV-4. A further neutralization test showed that of the four chimeric adenoviruses, only rAd3-A4R7-1 was neutralized by mouse anti-HAdV-4 serum (data not shown).

### Confirmation of the epitope recognized by MN4b using HAdV-4 HVR7 mutations.

**FIG 5:**
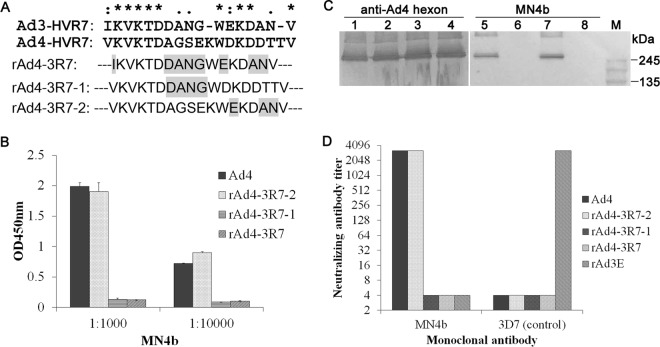
Confirmation of the epitope recognized by MN4b with mutations in HVR7 of HAdV-4. (A) The sequences shown represent HVR7 of HAdV-4 and HAdV-3 and the mutations in HVR7 of HAdV-4 hexon. Mutated residues are shown in gray. (B) Indirect ELISAs were performed to assess the reactions of serially diluted MN4b with HAdV-4 and HAdV-4 mutants containing chimeric hexon. (C) Immunoblot analyses of HAdV-4 (Ad4) (lanes 1 and 5), chimeric rAd4-3R7-1 (lanes 2 and 6), rAd4-3R7-2 (lanes 3 and 7), and rAd4-3R7 (lanes 4 and 8) were performed with MN4b (lanes 5 to 8) and antiserum from mice immunized with HAdV-4 hexon (lanes 1 to 4). (D) Neutralizing titers of MN4b that inhibited infection of AD293 cells by HAdV-4, rAd4-3R7-1, rAd4-3R-2, or rAd3E were determined by neutralization assays. MAb 3D7, a neutralizing antibody directed against HAdV-3, was used as a control. Antisera that showed no reactivity at a 1:16 dilution (the lowest dilution tested) were assigned a titer of 1:4.

HAdV-4 HVR7 can be divided into two independent regions, R7-1 and R7-2. To map the neutralizing epitope recognized by MN4b with an independent approach, three recombinant type 4 adenoviruses were generated, in which mutations were introduced into the long loop of HVR7 (Fig. 5A). These mutant viruses replicated to similar titers and showed similar particle/PFU ratios as those of the wild-type virus (data not shown), suggesting that mutations in R7 did not significantly alter the virion structure. These mutant viruses were then screened with MN4b by using an ELISA, Western blotting, and a neutralization test. A mutation in the R7-2 region (mutant rAd4-3R2) did not affect the recognition and neutralization of the recombinant virus by MN4b (Fig. 5). However, mutants rAd4-3R7-1 and rAd4-3R7, with altered R7-1 residues (AGSEK to DANG), escaped neutralization by MN4b (Fig. 5D). Data from ELISAs and Western blotting also indicated that rAd4-3R7-1 and rAd4-3R7 completely escaped recognition by MN4b (Fig. 5B and C). MAb 3D7, a neutralizing antibody against HAdV-3 prepared previously (33), was used as the control. These results confirm that R7-1 residues (AGSEK) form the critical epitope recognized by MN4b.

### R7 mutations reduce virus susceptibility to neutralization by HAdV-4 antisera.

**FIG 6:**
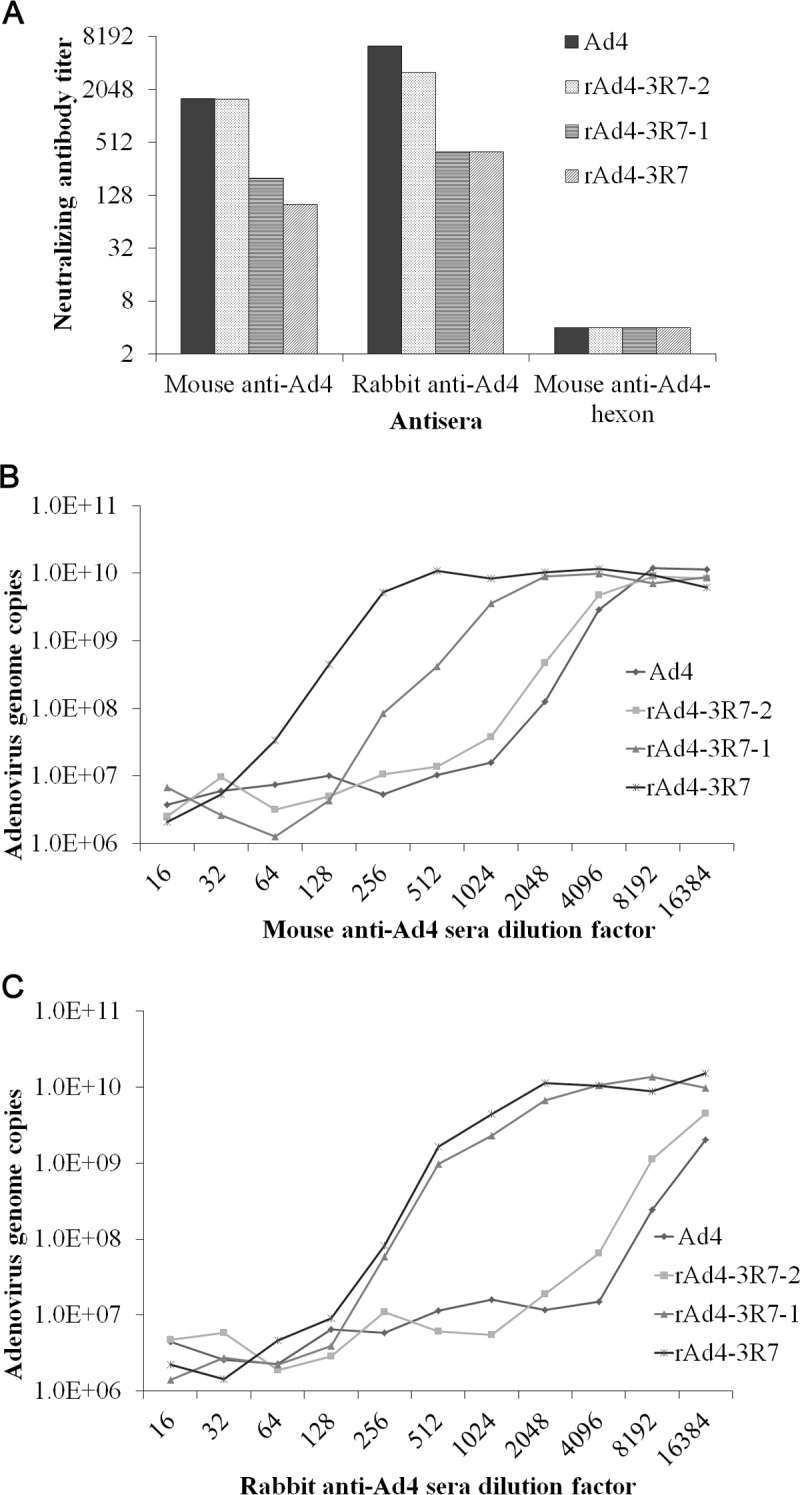
HAdV-4 R7-1 mutants partially escape neutralization by anti-HAdV-4 sera from mice and rabbits. (A) Neutralization of HAdV-4 mutants in AD293 cells by sera from HAdV-4-immunized mice or rabbits was measured by neutralization assays. Antisera that showed no reactivity at a 1:16 dilution (the lowest dilution tested) were assigned a titer of 1:4. (B and C) The neutralization assay results were confirmed by detection of the adenovirus genome copy numbers.

The susceptibility of R7 mutants to neutralization by anti-HAdV-4 serum was measured with neutralization tests. Pooled anti-HAdV-4 serum from mice showed 8-fold less neutralization activity against the rAd4-3R7-1 mutant and 16-fold less activity against the rAd4-3R7 mutant than against the wild-type virus. However, pooled anti-HAdV-4 serum from mice showed activity against rAd4-3R7-2 that was similar to that against wild-type HAdV-4. Similar results were observed with rabbit anti-HAdV-4 serum, which was about 16- to 32-fold less efficient in neutralizing the rAd4-3R7 and rAd4-3R7-1 mutants and about 2-fold less efficient in neutralizing rAd4-3R7-2 than in neutralizing the wild-type virus (Fig. 6). These results indicate that R7-1 contains a critical neutralization epitope, which accounts for most of the neutralizing antibody response to HAdV-4 in both mouse and rabbit.

**FIG 7:**
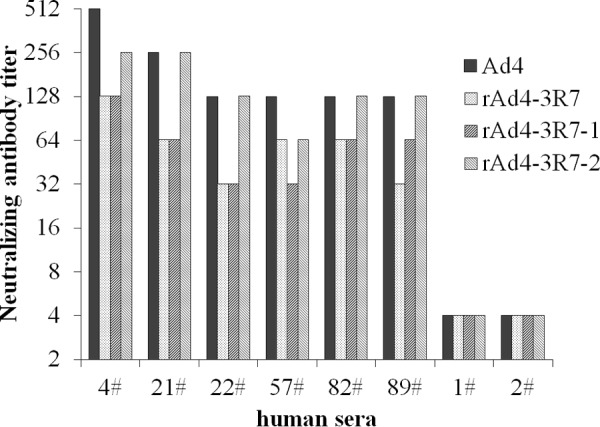
HAdV-4 R7-1 mutants partially escape neutralization by serum from humans infected with HAdV-4. Human serum samples 1 and 2, which showed no neutralization against HAdV-4, were used as negative controls. Sera that showed no reactivity at a 1:16 dilution (the lowest dilution tested) were assigned a titer of 1:4.

The susceptibility of the R7 mutants to neutralization by sera from humans infected with HAdV-4 was also tested. All six anti-HAdV-4-positive serum samples showed 2- to 4-fold less neutralization activity against rAd4-3R7-1 and rAd4-3R7 than against wild-type HAdV-4, whereas rAd4-3R7-2 showed similar susceptibility to all four anti-HAdV-4-positive serum samples as that of wild-type HAdV-4 (Fig. 7). These results demonstrate that R7-1 is a critical target of the neutralizing antibodies directed against HAdV-4 in human sera.

## DISCUSSION

In this study, we report a neutralizing MAb with high neutralizing activity against HAdV-4, which recognizes a conformational epitope (amino acid positions 418 to 422 in HAdV-4 hexon) within HVR7. This epitope is a critical target for NAbs directed against HAdV-4.

Previous crystallographic and phylogenetic analyses of hexon suggested that serotype-specific epitopes occur in the seven HVRs exposed on the virion surface (31). However, among the 52 known adenoviral serotypes, only a few neutralizing epitopes of HAdV-3, HAdV-7, HAdV-55, HAdV-14, and AdC68 have so far been identified, in experiments using a series of MAbs, synthetic peptides, or chimeric recombinant adenoviruses (28–30, 32–37). In this study, MN4b was shown to recognize a conformation-dependent epitope on the hexon trimer (Fig. 1). MN4b recognized only the R7-1 epitope but none of the other predicted epitopes of HAdV-4 that were displayed on HAdV-3 hexon (Fig. 3). However, MN4b reacted much more weakly with rAd3-A4R7-1 than with HAdV-4, which may be attributable to conformational differences in R7-1 when displayed on HAdV-4 hexon and HAdV-3 hexon. Mutations within the R7-1 epitope allowed recombinant HAdV-4 to completely escape neutralization by MN4b, confirming that MN4b recognizes the conformation-dependent R7-1 epitope (Fig. 5). Only rAd3-A4R7-1, but not rAd3-A4R1, rAd3-A4R2, or rAd3-A4R5, induced NAbs against HAdV-4 in mice, which suggests that of the four predicted sites, only R7-1 is an important neutralization epitope of HAdV-4 (Fig. 4).

Viruses with mutations within the R7-1 epitope were less susceptible than wild-type HAdV-4 to neutralization by anti-HAdV-4 serum from mice, rabbits, and humans (Fig. 6 and 7). These results suggest that R7-1 is the critical site in HAdV-4 for the induction of NAbs. We also found that mouse antiserum had a lower neutralizing titer against rAd4-3R7 than against rAd4-3R7-1 (Fig. 6), which indicates that other parts of R7 also affect neutralization. The great resistance of mutants with a few altered amino acids to neutralization highlights the need for continuous surveillance of HAdV-4 field strains.

Our data suggest that hexon is the predominant HAdV-4 antigen that induces NAbs, which is consistent with data from previous reports (25–27). However, HAdV-4 antisera still neutralized the mutated viruses. Therefore, further studies should investigate whether other epitopes within hexon or other capsid proteins, such as fiber or penton base, are also targets of NAbs in human sera.

**FIG 8:**
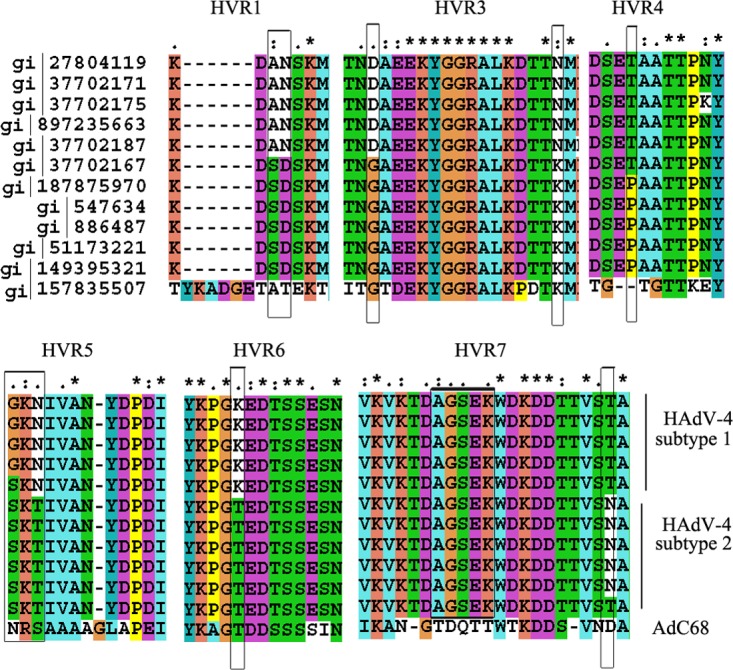
Multiple-sequence alignment of hexon proteins from HAdV-4 strains. The AdC68 sequence was included as a reference. Six hypervariable regions (HVR1 and HVR3 to -7) were identified. “*” indicates conserved amino acids, “.” indicates either conserved size or conserved hydropathy, and “:” indicates that both size and hydropathy are conserved. Gaps used to optimize the alignments are indicated by dashes. Variable sites between HAdV-4 subtypes are indicated by a gray frame.

MN4b recognizes HAdV-4 and inhibits infection by HAdV-4 but not HAdV-3 (Fig. 1). Therefore, MN4b may be used to distinguish HAdV-4 from other HAdV serotypes. An alignment of the hexon protein sequences available in GenBank revealed that the epitope recognized by MN4b is unique to HAdV-4. However, the distribution of this epitope among HAdV-4 strains, which might affect the detection and neutralization abilities of MN4b, must be determined. A phylogenetic analysis of the genomes and hexon sequences of global HAdV-4 strains available in GenBank showed that HAdV-4 can be classified into two subtypes (not shown here) and that some highly variable sites in the hexon protein vary between subtypes (Fig. 8). These sites were conserved within each of the subtypes. The epitope in R7-1 of different HAdV-4 strains (418AGSEK422) is highly conserved, and the sequences corresponding to this epitope are identical in both subtypes.

MN4b, which had a high neutralization titer of 3,200 (about 0.6 μg/ml), could potentially be humanized as a therapeutic or prophylactic agent against HAdV-4 infection (38). The identification of this important epitope may be useful for developing capsid-modified HAdV-4 vectors that would be permitted to escape preexisting antivector immunity (17, 20, 39).

The mechanism by which MN4b neutralizes HAdV-4 is unclear. It is also puzzling that HVR7-1 of HAdV-4 is a predominant epitope. Neutralization is defined as the reduction in viral infectivity caused by the binding of antibodies to the surfaces of viral particles (VPs), thereby blocking a step in the viral replication cycle that precedes virally encoded transcription or protein synthesis (40). HAdV infection is initiated by fiber binding, and the virus is then internalized via clathrin-coated vesicles after interactions occur between cellular integrins and penton base. An antibody may act at many steps of the adenovirus infection process (41). The interaction of MN4b with HAdV-4 hexon may block a step after virus entry, such as capsid disassembly, endosome penetration, translocation, or genome entry into the nucleus. Until now, the role of NAbs in inhibiting late steps in the entry process has been poorly characterized, although several studies have shown that an antihexon antibody inhibits adenoviral infection by blocking the microtubule-dependent translocation of the virus or by a TRIM21-dependent mechanism (42–44). The mechanism by which antihexon antibodies neutralize adenoviruses requires further investigation.

## MATERIALS AND METHODS

### Viral strains and cells.

Recombinant adenovirus rAd3EGFP (rAd3), which carries the HAdV-3 GZ-01 genome (GenBank accession no. DQ099432) and enhanced green fluorescent protein (eGFP) with an E3 region deletion, was generated as previously described (45). HAdV-4 strain GZ01 (GenBank accession no. KF006344.1) is maintained in our laboratory. Sublines of HEp-2 cells and AD293 cells are also kept in our laboratory and were cultured in Dulbecco's modified Eagle's medium (DMEM) (Gibco, USA) supplemented with penicillin (100 IU/ml), streptomycin (100 μg/ml), and 10% fetal bovine serum (Gibco) (25). All the adenoviruses were cultured in HEp-2 cells or AD293 cells, and adenoviral particles were purified with standard CsCl gradient centrifugation, as previously described (25). The VP titers were determined spectrophotometrically, using a conversion factor of 1.1 × 10^12^ VPs per absorbance unit at 260 nm (25).

### Defining the potential neutralizing epitope region of HAdV-4 hexon.

The primary amino acid sequence of HAdV-4 hexon was submitted to the SWISS-MODEL workspace for homology modeling. The SWISS-MODEL template library (SMTL version 2017-07-26, PDB release 2017-07-21) was searched with BLAST (46) and HHBlits (47) for evolutionarily related structures that matched the target sequence (48–50). The highest-quality templates were selected for model building. The models were built based on the target-template alignment using ProMod3. Coordinates that were conserved between the target and the template were copied from the template to the model. Insertions and deletions were remodeled by using a fragment library. Side chains were then rebuilt. Finally, the geometry of the resulting model was regularized with a force field. When loop modeling with ProMod3 failed, an alternative model was built with PROMOD-II (51). PyMOL v0.99 was used to generate the space-filling representation of the HAdV-4 hexon structure. The antigenic epitopes with lengths of 6 to 15 amino acids that were predicted to be exposed on the capsid surface and to be located in HVRs were selected as potential sites for recognition by NAbs.

### Generation of chimeric adenoviruses rAd3-A4R1, rAd3-A4R2, rAd3-A4R5, and rAd3-A4R7-1, which display the HAdV-4 epitopes on HAdV-3 hexon.

Plasmid pBRAd3dE3GFP, harboring the HAdV-3 GZ-01 genome (GenBank accession no. DQ099432) and eGFP with an E3 region deletion, was constructed as previously described (45). The shuttle vector pBRLR was also constructed as previously described (30). In this study, plasmids encoding HVR mutants (pBRAd3E-A4R1, pBRAd3E-A4R2, pBRAd3E-A4R5, and pBRAd3E-A4R7-1) were constructed with a strategy previously used to prepare plasmid rAdMHE1 (30). Briefly, the mutated fragment H3A4R1 was produced with overlapping PCR extension mutagenesis, using primer pairs A4HVR1u/HexD, A4HVR1r/HexU, and HexU/HexD and pBRAd3dE3GFP as the DNA template. The H3A4R1 fragment was then cloned into pBRLR to generate the shuttle vector pBRLR-H3A4R1. Finally, the LR-H3A4R1 fragment was cloned into the pBRAd3dE3GFP vector to generate the HAdV-3 hexon HVR1 mutagenesis vector pBRAd3E-A4R1, using homologous recombinant technology, in Escherichia coli strain BJ5183. These constructs were confirmed by restriction digestion and DNA sequencing analyses. To rescue the viruses, these plasmids were digested with AsiSI to linearize the genomic DNA and then used to transfect AD293 cells with Lipofectamine LTX with Plus reagent (Invitrogen, USA), according to the manufacturer's instructions. The transfected cells were cultured at 37°C under 5% CO_2_ for 6 to 10 days and were examined daily for evidence of a cytopathic effect (CPE). The cells were frozen and thawed for three cycles. The cell suspensions were collected and then used to infect the cells. At 96 h postinfection, the viruses were harvested and designated rAd3-A4R1, rAd3-A4R2, rAd3-A4R5, and rAd3-A4R7-1. Finally, the mutant viruses were cultured with AD293 cells in 20 dishes (100 mm), harvested, and purified with standard CsCl gradient centrifugation, as described above. The modified hexon genes of the viruses were confirmed by DNA sequencing.

### Generation of HAdV-4 hexon HVR7 mutants rAd4-A3R7, rAd4-A3R7-1, and rAd4-A3R7-2.

**FIG 9:**
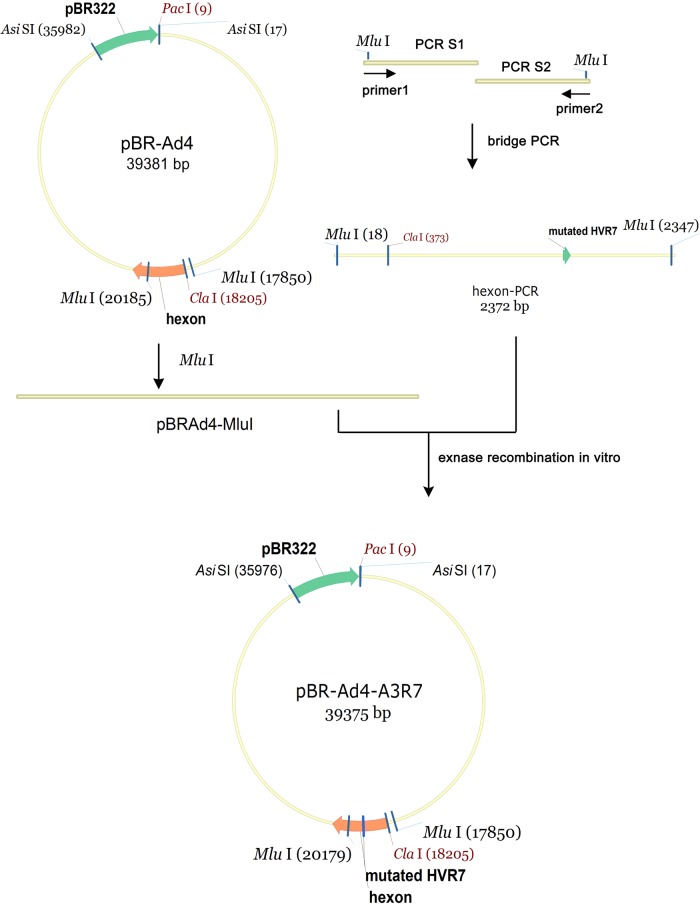
Introduction of a mutated site into the hexon gene in the HAdV-4 genome. Fragment H4A3R7 (here designated hexon-PCR), with a mutation in HVR7 of the HAdV-4 hexon region, was generated by overlapping PCR extension mutagenesis. The mutated fragment was then cloned into the MluI-digested pBRAd4 vector to generate the mutagenesis vector pBRAd4-A3R7 with homologous recombination *in vitro* by using Exnase II.

Plasmid pBRAd4 carrying the HAdV-4 GZ01 genome (GenBank accession no. KF006344.1) was constructed with a previously described strategy (45). Plasmids that carry the HVR mutants rAd4-A3R7, rAd4-A3R7-1, and rAd4-A3R7-2 were then constructed as shown in Fig. 9. Briefly, the mutated fragment H4A3R7 was produced by overlapping PCR extension mutagenesis with primer pairs A4-3R7-1u (5′-GTTAAAACAGATGACGCTAATGGATGGGACAAAGATGACACCACAG-3′)/Ad4-20204r (5′-CTCTTTGGTCTTGAGACGCGTGAAG-3′) and A4-3R7-1r (5′-TCATCTTTGTCCCATCCATTAGCGTCATCTGTTTTAACTTTAACACCCT-3′)/Ad4-17833u (5′-ACTGGGCCTGCCCACCACGCGTCCCA-3′), using pBRAd4 as the DNA template, and then with primer pair Ad4-17833u/Ad4-20204r. The H4A3R7 fragment was then cloned into the MluI-digested pBRAd4 vector to generate the mutagenesis vector pBRAd4-A3R7 with homology recombination *in vitro* with Exnase II (Vazyme, China). The vector was then transferred into E. coli for confirmation. Plasmids pBRAd4-A3R7-1 and pBRAd4-A3R7-2 were generated with the same method. The successful creation of these constructs was confirmed with restriction digestion and DNA sequencing analyses. Mutant viruses rAd4-A3R7, rAd4-A3R7-1, and rAd4-A3R7-2 were then rescued and purified in AD293 cells, as described above.

### Recombinant peptides and polyclonal antisera.

A recombinant HAdV-4 hexon peptide (amino acids 112 to 491 of hexon) expressed in E. coli and mouse antiserum directed against the HAdV-4 hexon peptide were prepared as described previously (52). Mouse antiserum against HAdV-3 was prepared as described previously (33). A recombinant HAdV-4 fiber knob peptide (amino acids 234 to 425 of fiber) was expressed in E. coli and purified with affinity chromatography using Ni-nitrilotriacetic acid (NTA) resin (Novagen, USA) under native conditions.

Rabbit antiserum was obtained from animals that had been injected intramuscularly with 5 × 10^11^ VPs of HAdV-4 and then boosted subcutaneously with the same dose after 3 weeks. Groups of five female BALB/c mice aged 4 to 6 weeks were immunized intramuscularly with 1 × 10^10^ VPs of HAdV-4, rAd3-A4R1, rAd3-A4R2, rAd3-A4R5, or rAd3-A4R7-1. Two booster doses were given at 2-week intervals with the same dose of antigen. Blood was collected from the anesthetized mice via the retro-orbital lobe 10 days after the final immunization, and the sera were isolated, heat inactivated, and stored frozen for serology tests.

The animal procedures used in this work were evaluated and approved by the Ethics Committee of the First Affiliated Hospital of Guangzhou Medical University (Guangzhou, China). They complied with all relevant guidelines and the National Law for Laboratory Animal Experimentation of China. The animal experiments were conducted in strict accordance with the recommendations of the *Guide for the Care and Use of Laboratory Animals* of the National Institutes of Health of United States (53). All animals were housed individually and received humane care. During injection and sample collection, the mice were anesthetized with 1.5% isoflurane or 1 ml/kg body weight of 3% pentobarbital sodium to minimize their suffering.

Serum samples from healthy human donors from Guangzhou (China) were collected after approval by the Ethics Committee of the First Affiliated Hospital of Guangzhou Medical University, and informed consent was obtained from each volunteer.

### Generation of NAbs.

Purified HAdV-4 virions were used to immunize BALB/c mice and to screen the resulting MAbs. The production and screening of mouse MAbs directed against HAdV-4 were performed as described previously (34). Briefly, BALB/c mice (6 to 8 weeks old) were injected intramuscularly with 1 × 10^10^ VPs of CsCl-purified HAdV-4 and then boosted twice intramuscularly with 5 × 10^9^ VPs of CsCl-purified HAdV-4 at 2-week intervals. Three days after intravenous boosting with 1 × 10^10^ VPs of HAdV-4 per mouse in phosphate-buffered saline (PBS), the mice were killed, and hybridoma fusion was performed by using a standard protocol (54). Hybridomas secreting HAdV-4-specific antibodies were screened with an ELISA with HAdV-4 virions. Positive hybridomas were then screened with a virus neutralization assay and subcloned with limiting dilution. Ascites were generated by injecting hybridoma cells into mice primed with Freund's incomplete adjuvant. The ascites titers against HAdV-4 were then determined with an ELISA, and the ascites were purified by octanoic acid-ammonium sulfate precipitation. The IgG concentrations were determined spectrophotometrically with a factor of 1.4 mg/ml per absorbance unit at 260 nm. The antibody isotypes were determined with a mouse hybridoma subtyping kit (Roche, Germany) according to the manufacturer's instructions.

### Indirect ELISA.

For ELISAs, 96-well Nunc MaxiSorp flat-bottom plates (Thermo, China) were coated with recombinant peptides (about 2 μg/ml) or virus particles (about 10^10^ VPs/ml) in PBS (pH 7.4) overnight at 4°C. They were then washed once with 0.05% Tween 20 in phosphate-buffered saline (PBST) and blocked for 2 h with 2% bovine serum albumin (BSA) in PBST. MAb ascites (100 μl/well) or antiserum in a series of dilutions from 1:100 to 1:1,000,000 was then added to each well and incubated for 1 h at 37°C. The plates were washed three times with PBST and incubated for 1 h with a 1:10,000 dilution of horseradish peroxidase (HRP)-conjugated goat anti-mouse IgG(H+L) affinity-purified secondary antibody or HRP-conjugated goat anti-rabbit IgG(H+L) secondary antibody. After the plates were washed four times with PBST, the products were visualized with the tetramethylbenzidine (TMB) substrate. The reaction was stopped with 1 M H_2_SO_4_, and the results were analyzed with an ELISA plate reader (Multiskan MK3; Thermo Scientific) at 450 nm.

### Virus neutralization test.

For *in vitro* adenovirus neutralization experiments, MAb ascites or antiserum pretreated at 56°C for 30 min was serially diluted 2-fold with DMEM (Gibco, China), and 50-μl aliquots of each dilution were mixed with an equal volume (100 50% tissue culture infective doses [TCID_50_]) of wild-type or recombinant adenovirus. The antibody-virus mixtures were incubated for 1 h at 37°C and transferred to 96-well plates containing 85% to 95% confluent monolayers of AD293 cells. After culture for 96 h, the monolayers were observed microscopically, and the neutralization titers were determined as the reciprocal of the highest dilution of mouse ascites or antiserum that protected the monolayer from a visually observable CPE. To confirm the results of the microneutralization assays, neutralization assays were performed under the same conditions, and the copies of the adenovirus genome were quantified with an adenovirus quantitative PCR (Q-PCR) kit (HYSMed, China) to measure the inhibition of viral infection by different sera (30).

### Immunoblot analysis.

AD293 cells were infected with wild-type adenovirus or the recombinant adenoviruses. At 72 h or 96 h postinfection, cells were harvested and freeze-thawed three times. The viral suspensions or purified virions were then mixed with 5× loading buffer (10% sodium dodecyl sulfate [SDS], 5% 2-mercaptoethanol, 0.5% bromophenol blue, and 50% glycerol in 250 mM Tris-HCl [pH 6.8]), kept at room temperature for 5 min, and then incubated on ice (native) or heated for 5 min at 98°C (denatured). The samples were then separated on 10% SDS-polyacrylamide gels and transferred electrophoretically onto polyvinylidene difluoride (PVDF) membranes. The membranes were blocked with 5% skim milk in PBS and then incubated with MAb ascites or mouse antiserum at final dilutions of 1:500 or 1:1,000. The membranes were washed again and exposed to a 1:10,000 dilution of HRP-conjugated goat anti-mouse IgG secondary antibody or HRP-conjugated goat anti-rabbit IgG secondary antibody. After washing, the blots were developed with the 1-Step Ultra TMB Blotting Solution substrate (Thermo Scientific, USA) for 5 min at room temperature.

## References

[1] Lenaerts L, De Clercq E, Naesens L 2008 Clinical features and treatment of adenovirus infections. Rev Med Virol 18:357–374. doi:10.1002/rmv.589. 18655013

[2] Sandkovsky U, Vargas L, Florescu DF 2014 Adenovirus: current epidemiology and emerging approaches to prevention and treatment. Curr Infect Dis Rep 16:416. doi:10.1007/s11908-014-0416-y. 24908344

[3] Stercz B, Nagy K, Ongradi J 2012 Adenovirus infections in immunocompromised patients. Orv Hetil 153:1896–1904. (In Russian.) doi:10.1556/OH.2012.29496. 23183005

[4] Dehghan S, Liu EB, Seto J, Torres SF, Hudson NR, Kajon AE, Metzgar D, Dyer DW, Chodosh J, Jones MS, Seto D 2012 Five genome sequences of subspecies B1 human adenoviruses associated with acute respiratory disease. J Virol 86:635–636. doi:10.1128/JVI.06593-11. 22158846 PMC3255920

[5] Fujimoto T, Yamane S, Ogawa T, Hanaoka N, Ogura A, Hotta C, Niwa T, Chiba Y, Gonzalez G, Aoki K, Koyanagi KO, Watanabe H 2014 A novel complex recombinant form of type 48-related human adenovirus species D isolated in Japan. Jpn J Infect Dis 67:282–287. doi:10.7883/yoken.67.282. 25056074

[6] Robinson CM, Singh G, Lee JY, Dehghan S, Rajaiya J, Liu EB, Yousuf MA, Betensky RA, Jones MS, Dyer DW, Seto D, Chodosh J 2013 Molecular evolution of human adenoviruses. Sci Rep 3:1812. doi:10.1038/srep01812. 23657240 PMC3648800

[7] Barrero PR, Valinotto LE, Tittarelli E, Mistchenko AS 2012 Molecular typing of adenoviruses in pediatric respiratory infections in Buenos Aires, Argentina (1999–2010). J Clin Virol 53:145–150. doi:10.1016/j.jcv.2011.11.001. 22138300

[8] Chen M, Zhu Z, Huang F, Liu D, Zhang T, Ying D, Wu J, Xu W 2015 Adenoviruses associated with acute respiratory diseases reported in Beijing from 2011 to 2013. PLoS One 10:e0121375. doi:10.1371/journal.pone.0121375. 25816320 PMC4376766

[9] Guo L, Gonzalez R, Zhou H, Wu C, Vernet G, Wang Z, Wang J 2012 Detection of three human adenovirus species in adults with acute respiratory infection in China. Eur J Clin Microbiol Infect Dis 31:1051–1058. doi:10.1007/s10096-011-1406-8. 21964587 PMC7087767

[10] Houng HS, Clavio S, Graham K, Kuschner R, Sun W, Russell KL, Binn LN 2006 Emergence of a new human adenovirus type 4 (Ad4) genotype: identification of a novel inverted terminal repeated (ITR) sequence from majority of Ad4 isolates from US military recruits. J Clin Virol 35:381–387. doi:10.1016/j.jcv.2005.11.008. 16406799

[11] Scott MK, Chommanard C, Lu X, Appelgate D, Grenz L, Schneider E, Gerber SI, Erdman DD, Thomas A 2016 Human adenovirus associated with severe respiratory infection, Oregon, USA, 2013-2014. Emerg Infect Dis 22:1044–1051. doi:10.3201/eid2206.151898. 27191834 PMC4880082

[12] Yun HC, Fugate WH, Murray CK, Cropper TL, Lott L, McDonald JM 2014 Pandemic influenza virus 2009 H1N1 and adenovirus in a high risk population of young adults: epidemiology, comparison of clinical presentations, and coinfection. PLoS One 9:e85094. doi:10.1371/journal.pone.0085094. 24416345 PMC3885690

[13] Gray GC, Goswami PR, Malasig MD, Hawksworth AW, Trump DH, Ryan MA, Schnurr DP 2000 Adult adenovirus infections: loss of orphaned vaccines precipitates military respiratory disease epidemics. For the Adenovirus Surveillance Group. Clin Infect Dis 31:663–670. doi:10.1086/313999. 11017812

[14] Hoke CH Jr, Snyder CE Jr 2013 History of the restoration of adenovirus type 4 and type 7 vaccine, live oral (adenovirus vaccine) in the context of the Department of Defense acquisition system. Vaccine 31:1623–1632. doi:10.1016/j.vaccine.2012.12.029. 23291475

[15] Kajon AE, Moseley JM, Metzgar D, Huong HS, Wadleigh A, Ryan MA, Russell KL 2007 Molecular epidemiology of adenovirus type 4 infections in US military recruits in the postvaccination era (1997-2003). J Infect Dis 196:67–75. doi:10.1086/518442. 17538885

[16] Radin JM, Hawksworth AW, Blair PJ, Faix DJ, Raman R, Russell KL, Gray GC 2014 Dramatic decline of respiratory illness among US military recruits after the renewed use of adenovirus vaccines. Clin Infect Dis 59:962–968. doi:10.1093/cid/ciu507. 24991024

[17] Alexander J, Ward S, Mendy J, Manayani DJ, Farness P, Avanzini JB, Guenther B, Garduno F, Jow L, Snarsky V, Ishioka G, Dong X, Vang L, Newman MJ, Mayall T 2012 Pre-clinical evaluation of a replication-competent recombinant adenovirus serotype 4 vaccine expressing influenza H5 hemagglutinin. PLoS One 7:e31177. doi:10.1371/journal.pone.0031177. 22363572 PMC3281928

[18] Roberts DM, Nanda A, Havenga MJ, Abbink P, Lynch DM, Ewald BA, Liu J, Thorner AR, Swanson PE, Gorgone DA, Lifton MA, Lemckert AA, Holterman L, Chen B, Dilraj A, Carville A, Mansfield KG, Goudsmit J, Barouch DH 2006 Hexon-chimaeric adenovirus serotype 5 vectors circumvent pre-existing anti-vector immunity. Nature 441:239–243. doi:10.1038/nature04721. 16625206

[19] Shiratsuchi T, Rai U, Krause A, Worgall S, Tsuji M 2010 Replacing adenoviral vector HVR1 with a malaria B cell epitope improves immunogenicity and circumvents preexisting immunity to adenovirus in mice. J Clin Invest 120:3688–3701. doi:10.1172/JCI39812. 20811151 PMC2947213

[20] Youil R, Toner TJ, Su Q, Chen M, Tang A, Bett AJ, Casimiro D 2002 Hexon gene switch strategy for the generation of chimeric recombinant adenovirus. Hum Gene Ther 13:311–320. doi:10.1089/10430340252769824. 11812286

[21] Gahery-Segard H, Farace F, Godfrin D, Gaston J, Lengagne R, Tursz T, Boulanger P, Guillet JG 1998 Immune response to recombinant capsid proteins of adenovirus in humans: antifiber and anti-penton base antibodies have a synergistic effect on neutralizing activity. J Virol 72:2388–2397. 9499099 10.1128/jvi.72.3.2388-2397.1998 PMC109538

[22] Roy S, Clawson DS, Calcedo R, Lebherz C, Sanmiguel J, Wu D, Wilson JM 2005 Use of chimeric adenoviral vectors to assess capsid neutralization determinants. Virology 333:207–214. doi:10.1016/j.virol.2004.12.029. 15721355

[23] Gall J, Kass-Eisler A, Leinwand L, Falck-Pedersen E 1996 Adenovirus type 5 and 7 capsid chimera: fiber replacement alters receptor tropism without affecting primary immune neutralization epitopes. J Virol 70:2116–2123. 8642632 10.1128/jvi.70.4.2116-2123.1996 PMC190048

[24] Sumida SM, Truitt DM, Lemckert AA, Vogels R, Custers JH, Addo MM, Lockman S, Peter T, Peyerl FW, Kishko MG, Jackson SS, Gorgone DA, Lifton MA, Essex M, Walker BD, Goudsmit J, Havenga MJ, Barouch DH 2005 Neutralizing antibodies to adenovirus serotype 5 vaccine vectors are directed primarily against the adenovirus hexon protein. J Immunol 174:7179–7185. doi:10.4049/jimmunol.174.11.7179. 15905562

[25] Tian X, Su X, Li H, Li X, Zhou Z, Liu W, Zhou R 2011 Construction and characterization of human adenovirus serotype 3 packaged by serotype 7 hexon. Virus Res 160:214–220. doi:10.1016/j.virusres.2011.06.017. 21740937

[26] Wu H, Dmitriev I, Kashentseva E, Seki T, Wang M, Curiel DT 2002 Construction and characterization of adenovirus serotype 5 packaged by serotype 3 hexon. J Virol 76:12775–12782. doi:10.1128/JVI.76.24.12775-12782.2002. 12438602 PMC136697

[27] Yu B, Dong J, Wang C, Zhan Y, Zhang H, Wu J, Kong W, Yu X 2013 Characteristics of neutralizing antibodies to adenovirus capsid proteins in human and animal sera. Virology 437:118–123. doi:10.1016/j.virol.2012.12.014. 23351390

[28] Bradley RR, Maxfield LF, Lynch DM, Iampietro MJ, Borducchi EN, Barouch DH 2012 Adenovirus serotype 5-specific neutralizing antibodies target multiple hexon hypervariable regions. J Virol 86:1267–1272. doi:10.1128/JVI.06165-11. 22072746 PMC3255845

[29] Pichla-Gollon SL, Drinker M, Zhou X, Xue F, Rux JJ, Gao GP, Wilson JM, Ertl HC, Burnett RM, Bergelson JM 2007 Structure-based identification of a major neutralizing site in an adenovirus hexon. J Virol 81:1680–1689. doi:10.1128/JVI.02023-06. 17108028 PMC1797575

[30] Qiu H, Li X, Tian X, Zhou Z, Xing K, Li H, Tang N, Liu W, Bai P, Zhou R 2012 Serotype-specific neutralizing antibody epitopes of human adenovirus type 3 (HAdV-3) and HAdV-7 reside in multiple hexon hypervariable regions. J Virol 86:7964–7975. doi:10.1128/JVI.07076-11. 22623776 PMC3421688

[31] Rux JJ, Kuser PR, Burnett RM 2003 Structural and phylogenetic analysis of adenovirus hexons by use of high-resolution X-ray crystallographic, molecular modeling, and sequence-based methods. J Virol 77:9553–9566. doi:10.1128/JVI.77.17.9553-9566.2003. 12915569 PMC187380

[32] Yuan X, Qu Z, Wu X, Wang Y, Liu L, Wei F, Gao H, Shang L, Zhang H, Cui H, Zhao Y, Wu N, Tang Y, Qin L 2009 Molecular modeling and epitopes mapping of human adenovirus type 3 hexon protein. Vaccine 27:5103–5110. doi:10.1016/j.vaccine.2009.06.041. 19573641

[33] Tian X, Liu M, Su X, Jiang Z, Ma Q, Liao X, Li X, Zhou Z, Li C, Zhou R 2015 Mapping the epitope of neutralizing monoclonal antibodies against human adenovirus type 3. Virus Res 208:66–72. doi:10.1016/j.virusres.2015.06.002. 26071383

[34] Liu M, Tian X, Li X, Zhou Z, Li C, Zhou R 2014 Generation of neutralizing monoclonal antibodies against a conformational epitope of human adenovirus type 7 (HAdv-7) incorporated in capsid encoded in a HAdv-3-based vector. PLoS One 9:e103058. doi:10.1371/journal.pone.0103058. 25054273 PMC4108376

[35] Ma Q, Tian X, Jiang Z, Huang J, Liu Q, Lu X, Luo Q, Zhou R 2015 Neutralizing epitopes mapping of human adenovirus type 14 hexon. Vaccine 33:6659–6665. doi:10.1016/j.vaccine.2015.10.117. 26546264

[36] Tian X, Li C, Xue C, Li X, Zhou Z, Zhou R 2013 Epitope mapping and characterization of a neutralizing monoclonal antibody against human adenovirus type 3. Virus Res 177:189–193. doi:10.1016/j.virusres.2013.08.013. 24018287

[37] Tian X, Ma Q, Jiang Z, Huang J, Liu Q, Lu X, Luo Q, Zhou R 2015 Identification and application of neutralizing epitopes of human adenovirus type 55 hexon protein. Viruses 7:5632–5642. doi:10.3390/v7102896. 26516903 PMC4632404

[38] Mair-Jenkins J, Saavedra-Campos M, Baillie JK, Cleary P, Khaw FM, Lim WS, Makki S, Rooney KD, Nguyen-Van-Tam JS, Beck CR, Convalescent Plasma Study Group. 2015 The effectiveness of convalescent plasma and hyperimmune immunoglobulin for the treatment of severe acute respiratory infections of viral etiology: a systematic review and exploratory meta-analysis. J Infect Dis 211:80–90. doi:10.1093/infdis/jiu396. 25030060 PMC4264590

[39] Appaiahgari MB, Vrati S 2015 Adenoviruses as gene/vaccine delivery vectors: promises and pitfalls. Expert Opin Biol Ther 15:337–351. doi:10.1517/14712598.2015.993374. 25529044

[40] Klasse PJ 2014 Neutralization of virus infectivity by antibodies: old problems in new perspectives. Adv Biol 2014:157895. doi:10.1155/2014/157895. 27099867 PMC4835181

[41] Wohlfart C 1988 Neutralization of adenoviruses: kinetics, stoichiometry, and mechanisms. J Virol 62:2321–2328. 3373570 10.1128/jvi.62.7.2321-2328.1988 PMC253388

[42] Smith JG, Cassany A, Gerace L, Ralston R, Nemerow GR 2008 Neutralizing antibody blocks adenovirus infection by arresting microtubule-dependent cytoplasmic transport. J Virol 82:6492–6500. doi:10.1128/JVI.00557-08. 18448546 PMC2447115

[43] Mallery DL, McEwan WA, Bidgood SR, Towers GJ, Johnson CM, James LC 2010 Antibodies mediate intracellular immunity through tripartite motif-containing 21 (TRIM21). Proc Natl Acad Sci U S A 107:19985–19990. doi:10.1073/pnas.1014074107. 21045130 PMC2993423

[44] McEwan WA, Hauler F, Williams CR, Bidgood SR, Mallery DL, Crowther RA, James LC 2012 Regulation of virus neutralization and the persistent fraction by TRIM21. J Virol 86:8482–8491. doi:10.1128/JVI.00728-12. 22647693 PMC3421726

[45] Zhang Q, Su X, Seto D, Zheng BJ, Tian X, Sheng H, Li H, Wang Y, Zhou R 2009 Construction and characterization of a replication-competent human adenovirus type 3-based vector as a live-vaccine candidate and a viral delivery vector. Vaccine 27:1145–1153. doi:10.1016/j.vaccine.2008.12.039. 19146906

[46] Altschul SF, Madden TL, Schaffer AA, Zhang J, Zhang Z, Miller W, Lipman DJ 1997 Gapped BLAST and PSI-BLAST: a new generation of protein database search programs. Nucleic Acids Res 25:3389–3402. doi:10.1093/nar/25.17.3389. 9254694 PMC146917

[47] Remmert M, Biegert A, Hauser A, Soding J 2011 HHblits: lightning-fast iterative protein sequence searching by HMM-HMM alignment. Nat Methods 9:173–175. doi:10.1038/nmeth.1818. 22198341

[48] Arnold K, Bordoli L, Kopp J, Schwede T 2006 The SWISS-MODEL workspace: a Web-based environment for protein structure homology modelling. Bioinformatics 22:195–201. doi:10.1093/bioinformatics/bti770. 16301204

[49] Benkert P, Biasini M, Schwede T 2011 Toward the estimation of the absolute quality of individual protein structure models. Bioinformatics 27:343–350. doi:10.1093/bioinformatics/btq662. 21134891 PMC3031035

[50] Biasini M, Bienert S, Waterhouse A, Arnold K, Studer G, Schmidt T, Kiefer F, Gallo Cassarino T, Bertoni M, Bordoli L, Schwede T 2014 SWISS-MODEL: modelling protein tertiary and quaternary structure using evolutionary information. Nucleic Acids Res 42:W252–W258. doi:10.1093/nar/gku340. 24782522 PMC4086089

[51] Guex N, Peitsch MC 1997 SWISS-MODEL and the Swiss-PdbViewer: an environment for comparative protein modeling. Electrophoresis 18:2714–2723. doi:10.1002/elps.1150181505. 9504803

[52] Tian X, Zhou R, Xue C, Li X, Zhou Z 2014 Cloning and expression of hexon protein of human respiratory adenovirus of three serotypes and analysis of antigenicity of the recombinant proteins. Chin J Microbiol Immunol 34:393–396. (In Chinese.)

[53] National Research Council. 2011 Guide for the care and use of laboratory animals, 8th ed National Academies Press, Washington, DC.

[54] Kohler G, Milstein C 2005 Continuous cultures of fused cells secreting antibody of predefined specificity. 1975. J Immunol 174:2453–2455. 15728446

